# Mass Spectrometry Imaging-Based Single-Cell Lipidomics Profiles Metabolic Signatures of Heart Failure

**DOI:** 10.34133/research.0019

**Published:** 2023-01-10

**Authors:** Jie Ren, Hao-Wen Li, Liang Chen, Min Zhang, Yan-Xiang Liu, Bo-Wen Zhang, Rui Xu, Yan-Yan Miao, Xue-Mei Xu, Xin Hua, Xiao-Gang Sun, Ru-Jia Yu, Yi-Tao Long, Sheng-Shou Hu

**Affiliations:** ^1^ State Key Laboratory of Cardiovascular Disease, Fuwai Hospital, National Center for Cardiovascular Disease, Chinese Academy of Medicine Science (CAMS) and Perking Union Medical College (PUMC), Beijing, 100037, P. R. China.; ^2^ Institute of Molecular Medicine, Shanghai Key Laboratory for Nucleic Acid Chemistry and Nanomedicine, State Key Laboratory of Oncogenes and Related Genes, Renji Hospital, School of Medicine, Shanghai Jiao Tong University, Shanghai, 200127, P. R. China.; ^3^ School of Chemistry and Molecular Engineering, East China University of Science and Technology, Shanghai, 200237, P. R. China.; ^4^ Jiangsu Engineering Laboratory of Smart Carbon-Rich Materials and Device, School of Chemistry and Chemical Engineering, Southeast University, Nanjing, 211189, P. R. China.; ^5^ State Key Laboratory of Analytical Chemistry for Life Science, School of Chemistry and Chemical Engineering, Nanjing University, Nanjing, 210023, P. R. China.

## Abstract

Heart failure (HF), leading as one of the main causes of mortality, has become a serious public health issue with high prevalence around the world. Single cardiomyocyte (CM) metabolomics promises to revolutionize the understanding of HF pathogenesis since the metabolic remodeling in the human hearts plays a vital role in the disease progression. Unfortunately, current metabolic analysis is often limited by the dynamic features of metabolites and the critical needs for high-quality isolated CMs. Here, high-quality CMs were directly isolated from transgenic HF mice biopsies and further employed in the cellular metabolic analysis. The lipids landscape in individual CMs was profiled with a delayed extraction mode in time-of-flight secondary ion mass spectrometry. Specific metabolic signatures were identified to distinguish HF CMs from the control subjects, presenting as possible single-cell biomarkers. The spatial distributions of these signatures were imaged in single cells, and those were further found to be strongly associated with lipoprotein metabolism, transmembrane transport, and signal transduction. Taken together, we systematically studied the lipid metabolism of single CMs with a mass spectrometry imaging method, which directly benefited the identification of HF-associated signatures and a deeper understanding of HF-related metabolic pathways.

## Introduction

Recent advances in the single-cell analysis have greatly enhanced the understanding of the cellular heterogeneity, which represents one of the most common features of dynamic cellular processes. The shift from bulk to single-cell studies could address the intrinsic heterogeneity of the cell population, which is masked in bulk measurements [1]. Such heterogeneity was well recognized to show the varieties of underlying disease pathogenesis and the rare subpopulation of cells [2]. For example, intratumor cellular heterogeneity, especially in genomics, has been clearly appreciated as the main factor for therapeutic resistance and was represented as one of the ongoing challenges in cancer therapy [3]. Therefore, the molecular profiling of the single cell is of fundamental importance in the field of bioscience. In recent years, single-cell omics including genomics, transcriptomics, proteomics, and metabolomics were surging to provide a detailed yet comprehensive molecular picture of individual cells [4]. Single-cell sequencing could identify some indiscoverable change of specific diseased cells, rather than the average of large populations of cells. In particular, metabolite acts as the final downstream product of biological activities [5], serving as the fundamental bridge between genotype and phenotype [6]. Mapping this single-cell metabolomics could produce an immediate and dynamic fingerprint of ongoing life activities and abnormal biological cellular processes [7]. However, single-cell metabolomics is still challenging in these omics measurements because of the dynamic and short-lived features of metabolite [8], which makes metabolomics to be known as the most immediate and direct indicator of cellular phenotype simultaneously [9].

Cardiovascular diseases remain the leading cause of mortality worldwide, urging a deeper understanding of the cardiovascular system. Etiopathogeny to date has been largely impeded by the complexity of the heart with regard to its cellular diversity [10]. Myocardial tissue is constitutive of multiple cell types including cardiomyocytes (CMs), fibroblasts, endothelial cells, smooth muscle cells, immune cells, and so on. Moreover, CMs also exhibit distinct heterogeneity of biochemistries. The heart is a high-energy-consuming organ that works uninterruptedly, with lipids acting as the main (70% to 90%) energy source for CMs. Previous studies have recognized the essential roles of metabolic remodeling and associated impaired signaling in the process of heart failure (HF) [11]. Metabolomics provides important insight into the cardiovascular disease by integrating genomic, transcriptomic, and environmental factors [12,13]. In addition, the cellular heterogeneity in CMs was the key to many mechanistic studies of cardiovascular pathophysiology [14]. Thus, single-cell metabolomics, particularly further lipid metabolism, was appreciated as a factor of structural and functional integrity of individual CMs [15,16]. Identification of abnormal lipid metabolism from individual CMs could help to understand the alterations in cellular signaling and function at an unprecedented resolution [17]. While CMs are connected via gap junctions and this structure is highly correlated with function, there are still difficulties in the isolation of high-quality CMs and maintenance of their typical morphologies [18]. Therefore, such a comprehensive and characteristic landscape of lipid metabolism in single CMs during HF has not been fully elucidated.

**Fig. 1.**
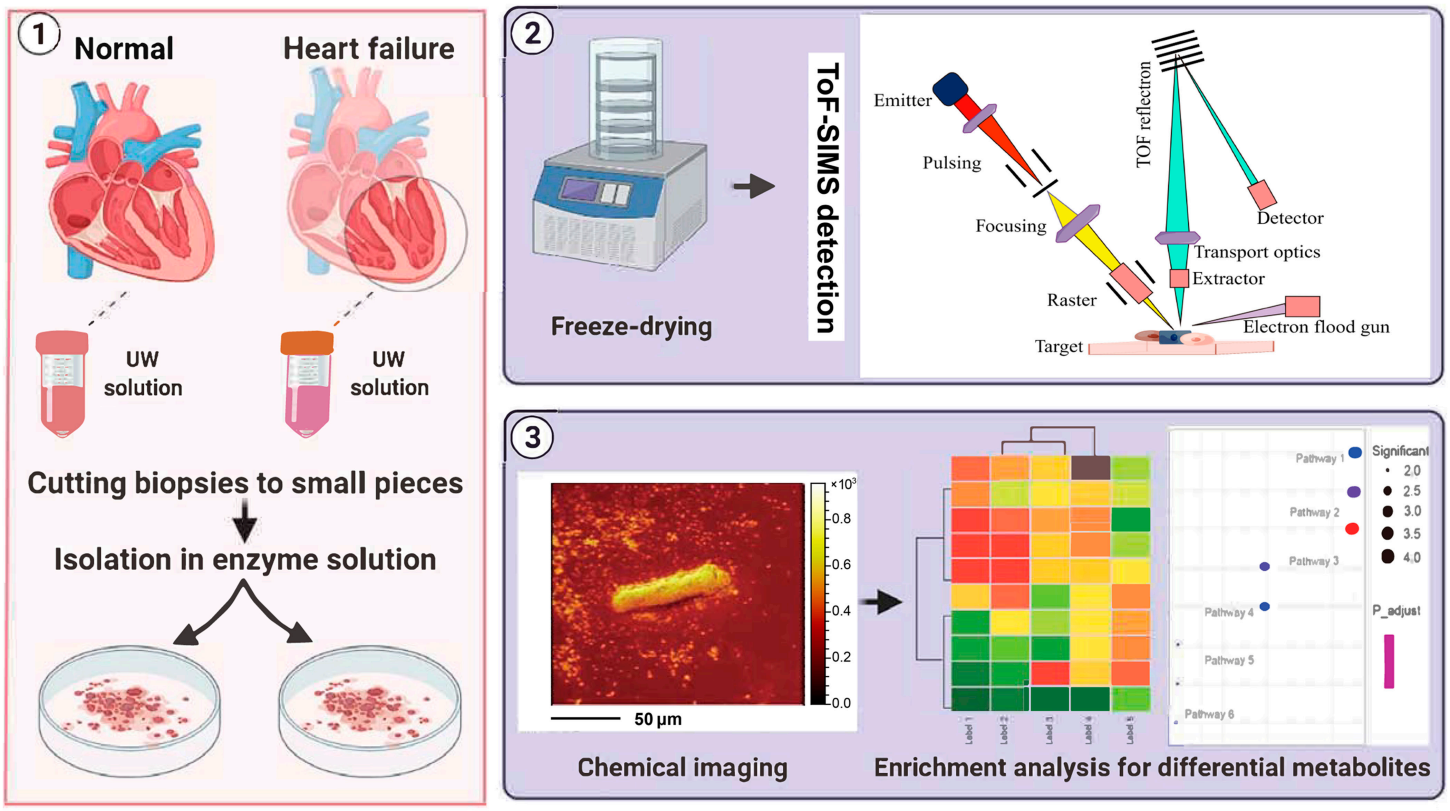
A method for lipidomics analysis in mammalian cardiomyocytes at a single-cell level.

Mass spectrometry has been widely used for metabolomics research due to its remarkable advantages of high sensitivity and high-throughput capability [19–21], which enables the simultaneous detection of a wide range of label-free biomolecules from complex samples [22–24]. It could be directly used to visualize the composition and spatial distribution of target molecules in individual cells [24–26]. Time-of-flight secondary ion mass spectrometry (ToF-SIMS) has been previously employed in the single-cell lipidomics with a high lateral resolution of ~200 nm and a depth resolution of ~1 nm [27]. In this study, by employing high-quality CMs isolated directly from biopsies for single-cell mass spectrometry, we profiled the dynamic composition and distribution of lipidomics in CMs during the HF process (Fig. 1). On the basis of the advantages of ToF-SIMS with high spatial resolution, lipid components could be obtained from different structures such as cell membrane and cell plasma to realize single-cell parametrization, phenotypic differentiation, and mechanistic investigation; meanwhile, molecular changes of diseases could be explored from different cell structure levels to explore the occurrence and development mechanism of diseases. High mass resolution and spatial resolution could be obtained simultaneously by applying the delayed extraction (DE) mode in ToF-SIMS. Here, we constructed an integrated method for single-cell lipidomics in CMs based on ToF-SIMS, which was further employed to identify characteristic HF-related metabolites. The HF-related metabolic pathways were further proposed, promoting a deep understanding of the disease mechanism and possible therapeutic targets.

## Results and Discussion

### Imaging and ToF-SIMS analysis of the isolated high-quality CMs

The CMs isolation here began with establishing a HF mice model, which developed typical morphological and functional phenotypes (Fig. S1). Specifically, echocardiography showed that HF mice exhibited dilated ventricles and dramatic decreases in systolic function. Further histopathology and immunostaining from HF mice’ heart tissues exhibited obvious myocardial fibrosis, cell apoptosis, and structural disarray. The detailed characterization of the HF mice model provided a reliable guarantee for subsequent isolation of high-quality CMs and single-cell lipidomics.

**Fig. 2.**
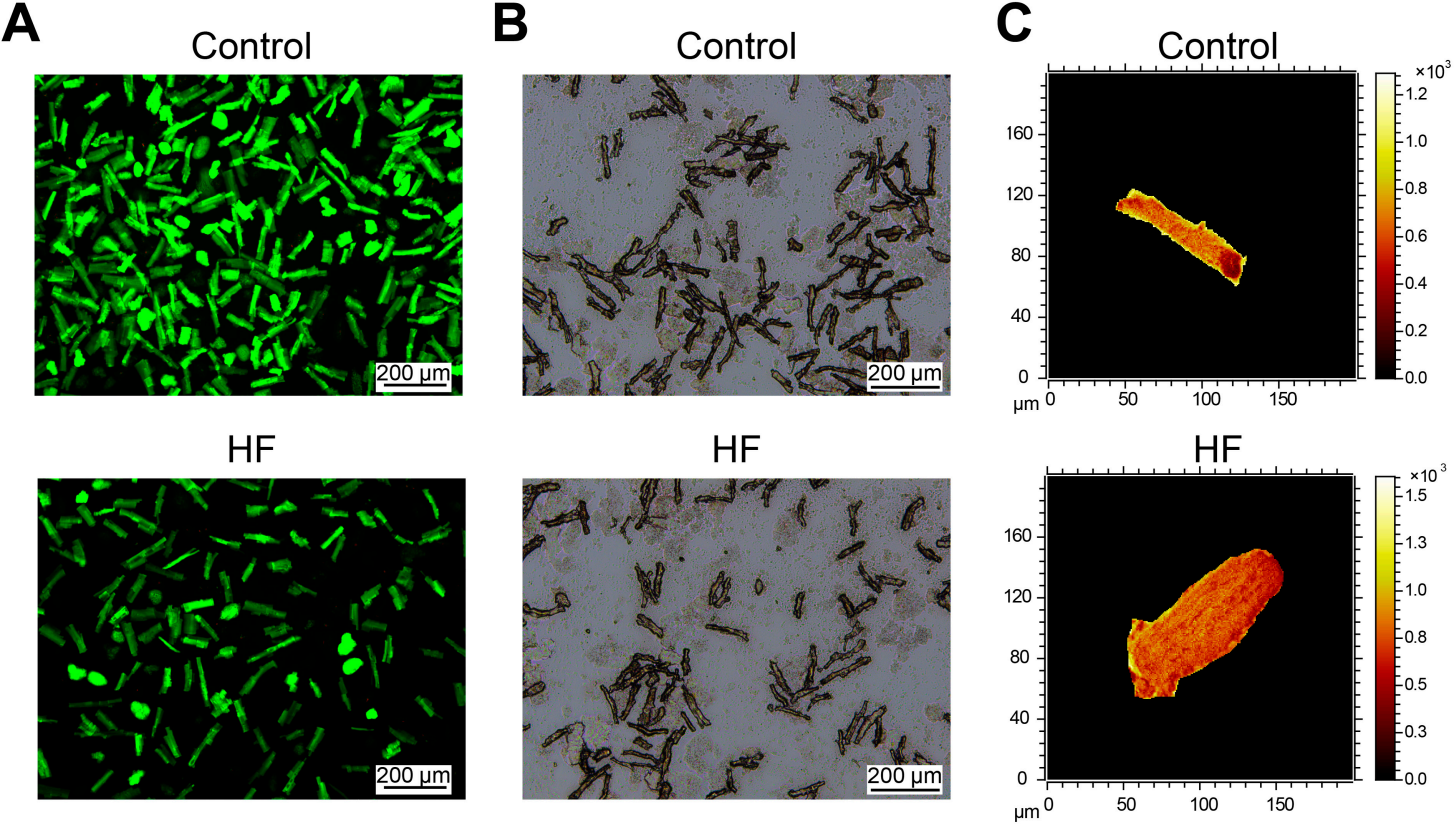
Images on the isolated mouse cardiomyocytes (CMs). (A) Immunofluorescence staining with calcein (green, live cells positive) of freshly isolated mouse CMs in heart failure (HF) and control groups. (B) Bright-field microscopy images on the CMs in HF and control groups. (C) Total secondary ions images on the CM samples at different groups.

Over the last decades, induced pluripotent stem cell (iPSC)-derived CMs have been generally adopted in cardiovascular studies [28], possibly because of the difficulties in isolating high-quality primary CMs [29]. However, the similarity of iPSC-derived CMs in morphology and function with fatal cells restrained its applications in late-onset diseases [30]. To date, the commonly used methods for isolating primary CMs have been enzymatic bulk digestion and the Langendorff method [30,31], both suffering from relatively low cell yield and the requirement of tissue integrity. By effectively combining tissue slicing and enzymatic digestion, the isolation approaches here robustly improved cell viability [32], preserving ionic reactions of CMs with intact morphology and function. Different imaging methods were employed for the detailed characterization of the freshly isolated CMs. First, fluorescent microscopy images of the isolated CMs were employed to characterize cell viability, which showed immunofluorescence staining with calcein and phalloidine (Fig. 2A and Fig. S1I). Detailed cell morphology was characterized by bright-field microscopy images, demonstrating that the isolated CMs maintained the typical rod-shaped morphology with an intact actin cytoskeleton (Fig. 2B and Fig. S1J). This isolation method could be confirmed to provide high-quality primary CMs, which exhibit normal morphological, functional, and molecular characteristics, and could reveal the pathophysiologic status in vivo. The total secondary ions images in Fig. 2C showed the capability of ToF-SIMS for single-cell imaging. Therefore, the superiority of ToF-SIMS for single-cell lipidomics lies in that it could first provide the morphology and phenotype for each tested CM, offering ample opportunities for exploring the relationship between its shape and function.

**Fig. 3.**
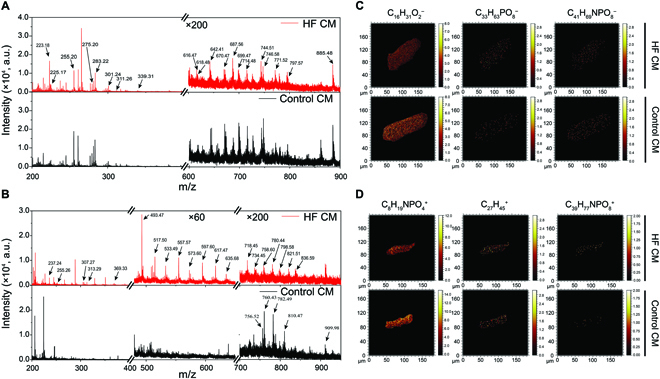
Overall lipid profiles of the single-cell mass spectrometry. (A) Negative and (B) positive ToF-SIMS spectra of CM under delayed extraction mode. For a clear display, the intensity of ion signal was amplified by different multiples. a.u., arbitrary units. (C and D) For the imaging components, including C_16_H_31_O_2_^−^ (FA 16:0, [M-H]^−^, m/z of 255.20), C_33_H_63_PO_8_^−^ (PC 28:0, [M-TMA]^−^, m/z of 618.48), C_41_H_69_NPO_8_^−^ (PE 36:6, [M-H]^−^, m/z of 734.47), C_8_H_19_NPO_4_^+^ (PC head group, m/z of 224.05), C_27_H_45_^+^ (cholesterol, [M-OH]^+^, m/z of 369.33), and C_39_H_77_NPO_8_^+^(PE34:1, [M+H]^+^, m/z of 718.45).

Full-scan mass spectra of single CM were acquired via ToF-SIMS at both the positive and negative ions modes, providing abundant molecular recognitions for cellular biomolecules. In the analysis process, 0.2 pA of Bi_3_^+^ ion beam in pulse mode and DE mode with 200-nm lateral resolution was employed as the analytical source, which is beneficial to obtain the quasi-molecular ion peaks of lipids components and to realize the molecular analysis of cell surface. A series of lipid fragments were observed in the ToF-SIMS spectra in the mass/charge ratio (m/z) range of 200 to 900. According to the previous study on lipid metabolism in coronary artery atherosclerosis, the lipid database was established with an error range of less than 100 ppm (parts per million) in the tissue analysis [33]. On the basis of these, the CM lipid database was constructed by respectively assigning specific mass peaks to corresponding positive and negative ions (Tables S1 and S2) [33]. Although ToF-SIMS could not obtain the precise structural information of lipids, it provides the quasi-molecular ion peaks of lipids in the mass range of 200 to 1000 Da and offers the information of carbon chain lengths and saturation degrees of lipids by the attribution of the mass peaks based on a TOF analyzer. It is beneficial for the relative quantitative analysis of the same lipid (with the same carbon chain lengths and saturation degrees) between different samples, which is currently accepted in the SIMS and matrix-assisted laser desorption/ionization mass spectrometry lipidomics analysis. Representative multiple fatty acids (FAs), phosphatidylethanolamines (PEs), sphingomyelins (SMs), phosphatidylinositols (PIs), and lysophospholipids were identified in negative mass spectra (Fig. 3A). Compared with control CMs, the signal intensities of m/z of 616.47, 618.48, 642.41, 699.47, 714.48, 720.47, 746.58, and 797.57 showed clear increases from the negative ion mass spectrum. Ion signals at m/z of 255.20, 618.48, and 734.47 were attributed to FA 16:0, PC 28:0, and PE 36:6, which were further employed for ToF-SIMS imaging. A clear lateral distribution of these characteristic lipid fragments inside HF and control CMs was shown in the 2D images. Meanwhile, a series of monoacylglycerides, diacylglycerides, phosphatidylcholines (PCs), triacylglycerides, and cholesterol were identified in positive mass spectra (Fig. 3B). Compared with control CMs, the signal intensity of m/z of 369.33, 493.47, 517.50, 533.49, 557.57, 573.60, 597.60, 617.47, and 718.45 showed a clear increase from positive ion mass spectrum. [M + H]^+^ ions at m/z of 224.05, 369.33, and 718.45 were assigned from the PC head group, cholesterol, and PE 34:1, respectively, and the corresponding 2D images showed the lipid distribution (Fig. 3C and D). Thus, the ToF-SIMS method could be employed for the identification of various lipid components in individual CM, with the imaging ability to visualize the distributions of specific ion fragments. Peak area of each lipid was normalized for further bioinformatics analysis due to the difficulties in direct identifying or further analyzing target lipid components among the mass spectra, especially for the species with relatively low abundance.

### Differential metabolomic analysis of the CMs

**Fig. 4.**
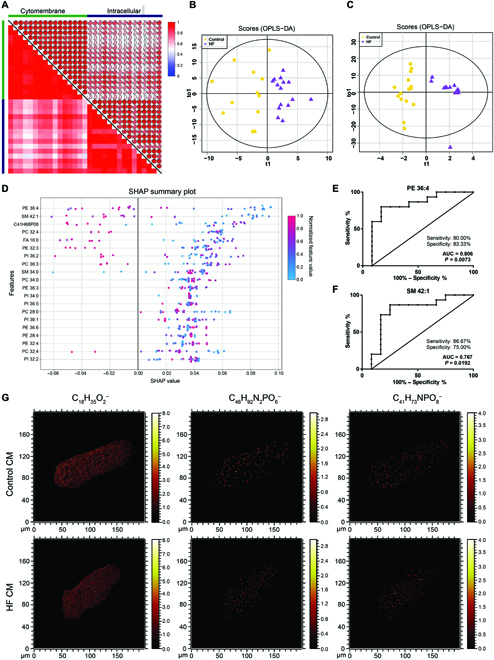
Differential metabolomic analysis of the CMs. (A) Canonical correlation analysis between the expression of detected metabolites of the cytomembrane and the intracellular in negative ion mass spectra. (B and C) OPLS-DA presented stark differences between the metabolites of the cytomembrane of 2 groups (yellow, normal control group; violet, HF group) (B) for the negative ion mass spectra and (C) for the positive ion mass spectra. (D) SHAP plot displayed variables in a top-to-bottom format to demonstrate the feature importance for prediction of HF in cytomembrane. (E and F) Receiver operating characteristic (ROC) curve to quantify the discriminability of metabolites to distinguish between the 2 groups (HF vs. control). AUC, area under the ROC curve. (G) ToF-SIMS imaging of CM’s surface of control and HF. C_18_H_35_O_2_^−^ is a characteristic fragment of FA 18:0, C_46_H_92_N_2_PO_6_^−^ is a characteristic fragment of SM 42:1, and C_41_H_73_NPO_8_^−^ is a characteristic fragment of PE 36:4.

High spatial resolution of ToF-SIMS enables the in-depth analysis of metabolic in single CMs. After 10 scans of a high-dose Ar_2000_^+^ sputter, the intracellular surfaces showed obvious charge accumulation. Thus, the images of the intracellular surfaces and the images of the cell membrane were obtained from 110 scans by the Bi_3_^+^ ion source. The mass spectra from the cell surface and intracellular surface were obtained separately for subsequent analysis. A total of 114 CMs were screened for profiling the composition and distribution of lipidomics (including both the positive and negative ions modes). The metabolic features of the cell surface were separated from those of the intracellular, which were illustrated by the canonical correlation analysis (Fig. 4A for the negative ion mass spectra and Fig. S2A for the positive ion mass spectra). This indicated that the degree of metabolic differences between the cell surface and intracellular surface was greater compared with those between HF and normal control. The discrimination between these groups could be facilitated by Orthogonal Partial Least Squares-Discriminant Analysis (OPLS-DA). The scores that are plotted in cell membrane metabolomics presented distinct differences between HF and control groups, at both negative and positive ion mass spectra (Fig. 4B and C). Likewise, intracellular metabolomics could also be discriminated between, as shown in Fig. S2B and Fig. 4C. The distinct difference in the single-cell metabolomics between the HF and control CMs, in particular, both in the cell membrane and intracellular surface indicates that HF CMs held unique metabolic expression profiles.

Then, further prediction method needs to be included for ranking variables. Previously, Student’s *t* test or Mann–Whitney test was generally used to predict features for the comparison of 2 conditions but could hardly be used for further applications in high-dimensional data because of the limitations in considering all confounding factors. Here, the SHAP (SHapley Additive exPlanation) method was adopted to rank the input variables according to their contribution to prediction [34]. SHAP calculated the feature importance for each single-cell spectrum instead of the overall difference between groups in the Student’s *t* test or Mann–Whitney test. In this way, the chemical heterogeneity revealed by single-cell lipidomics analysis could be preserved and the feature importance scores evaluated by SHAP-supervised analysis at a global scale are more comprehensive for our study. Therefore, the SHAP method was applied to analyze the influence values of the metabolites on the differential diagnosis of HF. In addition, the SHAP plot displayed metabolites in a top-to-bottom format, with the most important and least important metabolites displayed at the top and bottom, respectively, as shown in Fig. 4D and Fig. S2D. SHAP values (i.e., *x* axis) indicated the relative contribution of each cell’s metabolite to the overall prediction of HF, with negative values indicating the decreased predicted probability and positive values supporting the prediction of HF. It showed that PE 36:4 and SM 42:1 could be employed to discriminate between the cytomembrane of HF CMs and control CMs. Moreover, SM 34:0 and PC 30:1 could be used to distinguish intracellularly. By performing the receiver operating characteristic (ROC) curve, we further quantified the discriminability of those metabolites to distinguish HF from control groups (Fig. 4E and F and Fig. S2E and F). The results demonstrate that those differential metabolites could be regarded as efficient indicators/biomarkers for HF CMs (*P* < 0.05). Consistent with the metabolic profiles, the image features obtained from chemical mapping with high spatial resolution could also effectively differentiate the HF CMs from control CMs. As shown in Fig. 4G, the different components of the CM’s surface are SM 42:1 (C_46_H_92_N_2_PO_6_^−^) and PE 36:4 (C_41_H_73_NPO_8_^−^). On the cell membrane, FA 18:0 was selected as the internal reference rather than a different feature in SHAP plot because of the uniform distribution in both control CMs and HF CMs. According to the SHAP results, SM 42:1 and PE 36:4 with the highest differentiation contribution were decreased in HF CMs. There were aggregation signals in the local area of the cell membrane, especially PE 36:4. SM 34:0 (C_39_H_80_N_2_PO_6_^−^) and PC 30:1 (C_35_H_65_PO_8_^−^) were increased in the cytoplasm of HF CMs, which is showed in Fig. S2G. There were also aggregation signals in the local area of the cytoplasm. These results further confirm that ToF-SIMS can be used for elucidating the spatial distribution of metabolites in single cells.

These major phospholipids, including PE, SM, and PC, are the main building blocks for most biological membranes. PE is involved in multiple cellular functions and has a role in the membrane stability and function of membrane proteins [35]. PE appears to be playing a substantial function in the heart; the aggregation configuration of PE in CMs during myocardial ischemia induces membrane instability and cell damage [36]. SM is expressed predominately in the cytomembrane and endocytic recycling compartment [37], which is involved in functions such as the regulation of endocytosis and receptor-mediated ligand uptake, as well as G protein-coupled receptor function [38]. SM has also been mentioned in scientific literatures as an independent factor for the development of cardiovascular disease [38,39].

### Functional enrichment analysis for differential metabolites

**Fig. 5.**
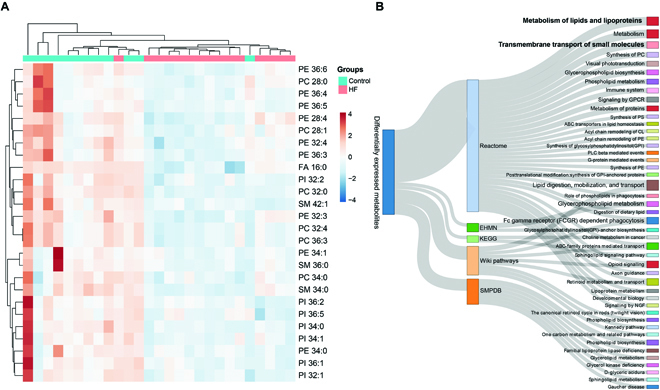
Functional implications of differential metabolites in the cytomembrane of CMs. (A) Hierarchical cluster analysis was used to visualize the clustering and relevance of the differential metabolites (aquamarine, normal control group; red, HF group). (B) Functional enrichment analysis of these differential metabolites.

We know that HF patients exhibited abnormal lipid metabolism, which has been implicated with disease progression [40]. However, previous studies investigating the relationships between lipidosis and HF have mainly focused on the tissue level. Lipids have high concentrations and important cellular functions, making them important metabolic targets for single-cell research [41]. The differentially expressed metabolites were first screened out by Student’s *t* test. Hierarchical cluster analysis was used to visualize the clustering and relevance of the differential metabolites, which were first screened out with *P* values of less than 0.05. As shown, there was a distinct decrease in the membrane expression of altered metabolites in the CMs of the HF group (Fig. 5A). The major species of these metabolites were found to be PI, PE, and PC. We performed pathway enrichment analysis using 5 different programs: REACTOME, EHMN, KEGG, WIKI, and SMPDB. These differential metabolites were mapped onto 45 pathways derived from the above databases. These reduced metabolites in cell surfaces of HF CMs were mainly enriched in pathways associated with lipid digestion/mobilization/transport, lipoprotein metabolism, and transmembrane transport of small molecules (Fig. 5B). These results suggested that the abnormal lipid molecules in HF CMs may be involved in HF development and progression through the above biological process and signaling pathways.

Cytomembrane makes a clear separation between inner (cytoplasmic) and outer (extracellular) compartments in cells, which provides a protective barrier and governs environmental energy resources, oxygen, and raw materials [42,43]. Decreases in lipid compositions would dramatically alter the physiochemical and biological characteristics of the cellular membrane, resulting in reduced membrane fluidity/viscosity, integrity, and permeability [44], and this change could result in a series of functional disorders of the heart and a variety of disease states [45]. Thus, it is reasonable to suspect that altered lipid composition/metabolism of cellular membranes provided characteristics for better metabolic targeting and intervention design for HF.

As an additional note, the intracellular metabolic differences were mainly reflected in the elevated levels of lipids in the HF CMs (predominantly lysophosphatidic acid, PI, and PE) (Fig. S3A). These obvious differences in the metabolic characteristics further specified the necessity of separate investigation for the cell surface and intracellular surface. Metabolic pathway enrichment analysis, performed in the MetaboAnalyst platform, indicated that elevated intracellular metabolites were related to lipids/lipoproteins metabolism and signal transduction (Fig. S3B). Previous studies have demonstrated that metabolic abnormalities and lipid deposition were shown in failing CMs. In addition, it implied a causal association between cardiac lipid overload and systolic/diastolic dysfunction [46]. Abnormal lipid metabolism was clearly associated with disease severity of HF with preserved ejection fraction (HFpEF) and was identified as the crucial pathophysiological driver of HFpEF in both preclinical models and clinical studies [47,48]. Metabolites are not only energy substrates for biological activities but also involved in the regulation of signaling cascades [49]. An imbalance in the lipid transport and lipid metabolism could induce CM death, cardiac injury, remodeling, and eventual cardiac dysfunction, which is known as lipotoxicity [50]. Lipotoxicity has been considered to play an essential role in the progression of HF. These toxic lipids or by-products have extensive impacts on the biological process of CMs [51]. Impairment of the intracellular metabolism in HF CMs would promote the lipid molecules into the lipotoxic pathway/signaling (Fig. S3), which further induced possible cardiac dysfunction.

Our results demonstrated that HF CMs displayed variations in lipids compositions both on the cellular surface and intracellular surface (Fig. 5). Therefore, we believed that the alteration in the lipids is a prominent feature of HF CMs, which may be involved in the pathogenesis of HF and targets for therapeutic intervention.

## Conclusion

In summary, we successfully integrated the cell isolation method with ToF-SIMS for single-cell lipidomics analysis in mammalian CMs. Different phenotypes of single CMs were profiled with a metabolic lipid panel based on the mass spectra. A series of metabolic features were identified and used for discrimination of the HF CMs from those of controls, which could be further used as HF biomarkers. HF-related metabolic pathways were revealed by pathway enrichment analysis, offering new opportunities for the disease mechanism and treatment. Therefore, this method provides crucial insights to discover efficient metabolites for disease investigation and search for potential therapeutic targets. Although single-cell lipidomics seems easily acquired by this ToF-SIMS imaging-based method, it is inherently hard to obtain a causal relationship. Further studies are still required to explore the disease relevance and mechanisms of these differences. Future development on the advanced mass spectrometry technologies would be helpful to further understand lipid structural information. In the future, this method could be used for precise disease analysis of clinical patient samples.

## Materials and Methods

### HF animal model

The C57BL/6 mice were purchased from Shanghai Model Organisms Centre Inc. (Shanghai, China) and maintained in plastic cages at 23 to 25 °C with a 12-hour light/12-hour dark cycle. A chronic HF model was established according to the previous literature procedure [52]. Renji Hospital’s Animal Ethics Committee approved the experimental protocol.

### Echocardiography

An ultrasound system (Vivid 7, USA) with a linear transducer of 13 MHz was used to perform echocardiographic experiments in mice at the age of 6 months [53]. An intraperitoneal injection of ketamine (50 to 100 mg/kg) was used to anesthetize the mice.

### Immunofluorescence staining

The tissue sections (10 μm thick) were fixed with methanol/acetone 1:1 for 20 min at −20 °C, after which the tissue was used for immunofluorescence analysis. Staining was performed using the following primary antibodies: sarcomere α-actinin (ab9465, Abcam, UK) and Cx43 (ab11370, Abcam, UK). Confocal images were taken using a Leica TCS SP5 DMI6000 inverted microscope (with the TCS SP5 confocal scanner and acquisition software LAS-AF; Leica Microsystems, Mannheim, Germany). The Zeiss AxioScan Z1 digitized the immunofluorescence images.

### Western blot for protein analysis

The procedures were conducted as previously reported in cardiac tissue extracts, except the other way indicated. The antibodies included are the following: Natriuretic peptides A (NPPA) (ab225844, Abcam, UK) and glyceraldehyde phosphate dehydrogenase (GAPDH) (ab8245, Abcam, UK). GAPDH was used as the internal reference of the protein expression.

### RT-qPCR for gene expression

The relative gene expression levels in cardiac samples were detected using a SYBR Green-based polymerase chain reaction (PCR) kit (Applied Biosystems, Foster City, CA, USA). The specific protocols for RNA extraction and reverse transcription were described in previous study. The *Gapdh* was used as the internal reference gene of the reverse transcription quantitative PCR (RT-qPCR). The ΔΔCt method was used for the calculation of relative gene expression levels. The following validated primers (Nppa, Mm01255747_g1, TaqMan, Applied Biosystems) were used.

### Isolation of mice CMs

CMs were isolated as previously described [32]. After the mice hearts were taken, blood was immediately removed, and the samples were immediately placed in precooling University of Wisconsin (UW) solution for tissue transport. Then, the myocardium was sliced into 200-μm-thick tissue slices using steel blades with an advance rate of 0.3 mm/s and a vibration of 2.5 mm in amplitude. The tissue slices were further triturated and transferred to an enzyme solution containing 275 U/ml of collagenase type II (Worthington) and 1.2 U/ml of protease (type X X IV, Sigma). Then, we swirl the enzyme solution around the flask gently and incubate at 37 °C. When the supernatant firstly became turbid, the supernatant was removed and discarded. The tissue chunks enzymatically redigested in the fresh enzyme solution (same recipe as above). The CMs suspension was obtained by filtering the digestion mixture through 100-μm filters and centrifuging the samples (100g). This study was approved by the Ethics Committee of Renji Hospital, Shanghai, China.

### Preparation of ITO coverslips and cell samples

The indium tin oxide (ITO) coverslips were ultrasonic cleaned in acetone, ethanol, and pure water for 0.5 h, successively, and then washed by piranha solution (*V*_H2SO4_:*V*_H2O2_ = 3:1). Subsequently, the treated ITO coverslips were washed using pure water and dried with nitrogen. Meanwhile, the above single-cell suspension was centrifuged and then resuspended with 150 mM ammonium acetate solution 3 times. The single-cell suspension was dropped on the ITO coverslips. A Kimwipe paper was used to blot the remaining ammonium acetate solution from the edge of the ITO coverslips. CMs attached on the ITO coverslips were then fast frozen in liquid nitrogen at −196 °C to maintain the cells’ shape before being introduced to the vacuum freeze drier. The CM samples were lyophilized for 6 h.

### ToF-SIMS instrumentation

All ToF-SIMS surface analyses were measured using a 30-keV Bi_3_^+^ primary ion beam as analytical source, depth profiles were measured using a 10-keV Ar_2000_^+^ sputter gun, and charge compensation was performed using a 20-eV electron flood gun. Mass spectra and chemical mapping of single cells were recorded by DE mode. Under DE mode, the target current of Bi_3_^+^ ion beam was ~0.2 pA on faraday cup, and the optimized lateral resolution was about 200 nm on 1000 mesh. Meanwhile, the mass resolution (*m/Δm*) was 5600 at m/z of 184.04 (C_5_H_15_NPO_4_^+^). Depth profiling of cells was used in noninterlaced mode. The incident angle was 45° for the Bi_3_^+^ ion beam, Ar_2000_^+^ ion beam, and electron beam. The included angle was ~31° between the Bi_3_^+^ ion beam and electron beam and 60° between the Bi_3_^+^ ion beam and Ar_2000_^+^ ion beam. All cell surfaces images were obtained from 110 scans by Bi_3_^+^ primary ion beam. Then, the intracellular surfaces of all cells were obtained from 10 scans by an Ar_2000_^+^ sputter gun. All intracellular surfaces images were obtained from 110 scans by a Bi_3_^+^ primary ion beam.

### ToF-SIMS data analysis

ToF-SIMS spectra and chemical mapping were analyzed by SurfaceLab 7.1 software. Single-cell areas were selected using region of interest analysis. Signals in the mass range of 400 to 900 Da were amplified by 60 to 200 times overall, leading to the signal strength of mass peaks that was ~1 × 10^4^ magnitude (arbitrary units). There was a comparable signal distribution on the cell membrane surface and cytomembrane of CMs, and the signal intensity of the mass spectral peaks implicated in the study was all 3 times that of the baseline. All data used for quantification were recorded under static limit. C_2_H_5_^+^, C_5_H_12_N^+^, C_5_H_13_NPO_3_^+^, and C_5_H_15_NPO_4_^+^ were chosen to calibrate all positive ToF-SIMS spectra, while C_2_^−^, C_3_^−^, C_4_^−^, and C_4_H^−^ were chosen to calibrate all negative spectra.

### Statistical analysis

Categorical variables were analyzed by chi-square tests or Fisher’s exact test. For continuous variables, the *t* test or Mann–Whitney U test was used for comparisons between 2 groups, and the Kruskal-Wallis test was used for comparisons among multiple groups. The ROC curve and area under the ROC curve analyses were plotted to describe the prediction accuracy of specific metabolites for HF. Statistical analyses were performed using SPSS Statistics, version 23.0 (IBM Corporation, Armonk, NY, USA), and R version 4.0.0. Statistical charts were plotted using GraphPad Prism 7 (GraphPad Software Inc., CA, USA) and ggplot2 (version 3.3.3). Pathway enrichment analysis of the differential metabolites was conducted using the MetaboAnalyst 5.0. The SHAP method was applied by executing package “shap”’ to analyze feature importance for prediction of HF. Covariates with *P* < 0.05 were considered significant.

## Data Availability

All data supporting the findings of this study are available within the paper and its Supplementary Materials.

## Supplementary Materials

Supplementary Materials Table S1. Peak assignment of ToF-SIMS spectra of CMs to corresponding negative ions. Table S2. Peak assignment of ToF-SIMS spectra of CMs to corresponding positive ions. Fig. S1. Characterization of the HF mouse model. Fig. S2. Differential metabolomic analysis of the CMs. Fig. S3. Functional implications of intracellular differential metabolites of CMs.
