# Supplementary material for: Mass Spectrometry Imaging-Based Single-Cell Lipidomics Profiles Metabolic Signatures of Heart Failure
Source: Research (Wash D C). 2023 Jan 10;6:0019. doi: 10.34133/research.0019 (PMC10076023; doi:10.34133/research.0019)
Supplement: Supplementary Materials — Table S1. Peak assignment of ToF-SIMS spectra of CMs to corresponding negative ions. Table S2. Peak assignment of ToF-SIMS spectra of CMs to corresponding positive ions. Fig. S1. Characterization of the HF mouse model. Fig. S2. Differential metabolomic analysis of the CMs. Fig. S3. Functional implications of intracellular differential metabolites of CMs. [file research.0019.f1.pdf]

Supporting Information

Mass spectrometry imaging-based single-cell lipidomics profiles metabolic signatures of heart failure

Jie Ren[1]†, Hao-Wen Li[2,4]†, Liang Chen[1]†, Min Zhang[4], Yan-Xiang Liu[1], Bo-Wen Zhang[1], Rui Xu[2], Yan-Yan Miao[2], Xue-Mei Xu[2], Xin Hua[4,5], Xiao-Gang Sun[1], Ru-Jia Yu[3,4]*, Yi-Tao Long[3,4]*, Sheng-Shou Hu[1]*

[1.] State Key Laboratory of Cardiovascular Disease, Fuwai Hospital, National Center for Cardiovascular Disease, Chinese Academy of Medicine Science (CAMS) and Perking Union Medical College (PUMC), Beijing, 100037, P. R. China
[2.] Institute of Molecular Medicine, Shanghai Key Laboratory for Nucleic Acid Chemistry and Nanomedicine, State Key Laboratory of Oncogenes and Related Genes, Renji Hospital, School of Medicine, Shanghai Jiao Tong University, Shanghai, 200127, P. R. China
[3.] State Key Laboratory of Analytical Chemistry for Life Science, School of Chemistry and Chemical Engineering, Nanjing University, Nanjing, 210023, P. R. China
[4.] School of Chemistry and Molecular Engineering, East China University of Science and Technology, Shanghai, 200237, P. R. China
[5.] Jiangsu Engineering Laboratory of Smart Carbon-Rich Materials and Device, School of Chemistry and Chemical Engineering, Southeast University, Nanjing, 211189, P. R. China

†These authors contributed equally to this work

*Correspondence should be addressed to Ru-Jia Yu; yurujia@nju.edu.cn, Yi-Tao Long; yitaolong@nju.edu.cn and Sheng-Shou Hu; huss@fuwaihospital.org.

Table S1. Peak assignment of ToF-SIMS spectra of cardiomyocytes to corresponding negative ions

No.	Center Mass (u)	Assignment	Description	Mass Deviation (ppm)
1	223.18	$C_{14}H_{23}O_2^-$	FA14:2 $[M-H]^-$	31.3
2	225.17	$C_{14}H_{25}O_2^-$	FA14:1 $[M-H]^-$	-85.6
3	227.18	$C_{14}H_{27}O_2^-$	FA14:0 $[M-H]^-$	-99.4
4	249.20	$C_{16}H_{25}O_2^-$	FA16:3 $[M-H]^-$	40.6
5	251.19	$C_{16}H_{27}O_2^-$	FA16:2 $[M-H]^-$	-49.3
6	253.19	$C_{16}H_{29}O_2^-$	FA16:1 $[M-H]^-$	-110.9
7	255.20	$C_{16}H_{31}O_2^-$	FA16:0 $[M-H]^-$	-138.9
8	275.20	$C_{18}H_{27}O_2^-$	FA18:4 $[M-H]^-$	2.3
9	277.20	$C_{18}H_{29}O_2^-$	FA18:3 $[M-H]^-$	-49.6
10	279.19	$C_{18}H_{31}O_2^-$	FA18:2 $[M-H]^-$	-137.5
11	281.21	$C_{18}H_{33}O_2^-$	FA18:1 $[M-H]^-$	-139.1
12	283.22	$C_{18}H_{35}O_2^-$	FA18:0 $[M-H]^-$	-138.7
13	301.24	$C_{20}H_{29}O_2^-$	FA20:5 $[M-H]^-$	67.3
14	303.20	$C_{20}H_{31}O_2^-$	FA20:4 $[M-H]^-$	-114.3
15	305.23	$C_{20}H_{33}O_2^-$	FA20:3 $[M-H]^-$	-52.4
16	307.24	$C_{20}H_{35}O_2^-$	FA20:2 $[M-H]^-$	-70.1
17	309.25	$C_{20}H_{37}O_2^-$	FA20:1 $[M-H]^-$	-80.6
18	311.26	$C_{20}H_{39}O_2^-$	FA20:0 $[M-H]^-$	-116.1
19	327.23	$C_{22}H_{31}O_2^-$	FA22:6 $[M-H]^-$	-6.9
20	329.23	$C_{22}H_{33}O_2^-$	FA22:5 $[M-H]^-$	-61.8
21	331.23	$C_{22}H_{35}O_2^-$	FA22:4 $[M-H]^-$	-115.3
22	333.26	$C_{22}H_{37}O_2^-$	FA22:3 $[M-H]^-$	-56.0
23	335.27	$C_{22}H_{39}O_2^-$	FA22:2 $[M-H]^-$	-83.4
24	337.29	$C_{22}H_{41}O_2^-$	FA22:1 $[M-H]^-$	-51.3
25	339.31	$C_{22}H_{43}O_2^-$	FA22:0 $[M-H]^-$	-48.2

26	614.43	$C_{33}H_{59}PO_8^-$	PC28:2 [M-TMA]$^-$	55.1
27	616.48	$C_{33}H_{61}PO_8^-$	PC28:1 [M-TMA]$^-$	115.1
28	618.48	$C_{33}H_{63}PO_8^-$	PC28:0 [M-TMA]$^-$	92.5
29	626.41	$C_{33}H_{57}NPO_8^-$	PE28:4 [M-H]$^-$	42.6
30	628.48	$C_{33}H_{59}NPO_8^-$	PE28:3 [M-H]$^-$	126.6
31	630.43	$C_{33}H_{61}NPO_8^-$	PE28:2 [M-H]$^-$	25.3
32	632.34	$C_{33}H_{63}NPO_8$-	PE28:1 [M-H]-	-145.0
33	634.54	$C_{33}H_{65}NPO_8$-	PE28:0 [M-H]-	151.3
34	640.47	$C_{35}H_{61}PO_8^-$	PC30:3 [M-TMA]$^-$	97.5
35	642.41	$C_{35}H_{63}PO_8^-$	PC30:2 [M-TMA]$^-$	-27.7
36	644.47	$C_{35}H_{65}PO_8^-$	FA 16:0 [M-TMA]$^-$	45.7
37	646.50	$C_{35}H_{67}PO_8^-$	PC30:0 [M-TMA]$^-$	61.5
38	654.42	$C_{35}H_{61}NPO_8^-$	PE30:4 [M-H]$^-$	9.6
39	656.46	$C_{35}H_{63}NPO_8^-$	PE30:3 [M-H]$^-$	44.2
40	658.44	$C_{35}H_{65}NPO_8^-$	PE30:2 [M-H]$^-$	-12.1
41	660.42	$C_{35}H_{67}NPO_8^-$	PE30:1 [M-H]$^-$	-67.1
42	662.52	$C_{35}H_{69}NPO_8^-$	PE30:0 [M-H]$^-$	68.6
43	666.42	$C_{37}H_{63}PO_8^-$	PC32:4 [M-TMA]$^-$	-4.9
44	668.48	$C_{37}H_{65}PO_8^-$	PC32:3 [M-TMA]$^-$	54.7
45	670.46	$C_{37}H_{67}PO_8^-$	PC32:2 [M-TMA]$^-$	4.2
46	672.49	$C_{36}H_{67}NPO_8^-$	PC28:2 [M-H]$^-$	49.8
47	672.49	$C_{37}H_{69}PO_8^-$	PC32:1 [M-TMA]$^-$	31.1
48	674.40	$C_{36}H_{69}NPO_8^-$	PC28:1 [M-H]$^-$	-114.7
49	674.40	$C_{37}H_{71}PO_8^-$	PC32:0 [M-TMA]$^-$	-133.4
50	676.41	$C_{36}H_{71}NPO_8^-$	PC28:0 [M-H]$^-$	-124.9
51	682.47	$C_{37}H_{65}NPO_8^-$	PE32:4 [M-H]$^-$	42.2
52	684.45	$C_{37}H_{67}NPO_8^-$	PE32:3 [M-H]$^-$	-12.5
53	685.50	$C_{38}H_{74}N_2PO_6^-$	SM34:2(16:2) [M-CH3]$^-$	-48.3
54	686.45	$C_{37}H_{69}NPO_8^-$	PE32:2 [M-H]$^-$	-37.6

55	687.56	$C_{38}H_{76}N_2PO_6^-$	SM34:1(16:1) [M-CH3]$^-$	24.8
56	688.59	$C_{37}H_{71}NPO_8^-$	PE32:1 [M-H]$^-$	137.8
57	689.53	$C_{38}H_{78}N_2PO_6^-$	SM34:0(16:0) [M-CH3]$^-$	-44.9
58	690.46	$C_{37}H_{73}NPO_8^-$	PE32:0 [M-H]$^-$	-67.8
59	692.42	$C_{39}H_{65}PO_8^-$	PC34:5 [M-TMA]$^-$	-38.6
60	694.53	$C_{39}H_{67}PO_8^-$	PC34:4 [M-TMA]$^-$	98.7
61	696.44	$C_{39}H_{69}PO_8^-$	PC34:3 [M-TMA]$^-$	-44.2
62	698.44	$C_{38}H_{69}NPO_8^-$	PC30:3 [M-H]$^-$	-48.4
63	698.44	$C_{39}H_{71}PO_8^-$	PC34:2 [M-TMA]$^-$	-66.4
64	699.47	$C_{39}H_{76}N_2PO_6^-$	SM34:2(16:2) [M-H]$^-$	-113.5
65	700.45	$C_{38}H_{71}NPO_8^-$	PC30:2 [M-H]$^-$	-67.1
66	700.45	$C_{39}H_{73}PO_8^-$	PC34:1 [M-TMA]$^-$	-85.1
67	701.48	$C_{39}H_{78}N_2PO_6^-$	SM34:1(16:1) [M-H]$^-$	-111.8
68	702.54	$C_{38}H_{73}NPO_8^-$	PC30:1 [M-H]$^-$	39.7
69	702.54	$C_{39}H_{75}PO_8^-$	PC34:0 [M-TMA]$^-$	21.8
70	703.49	$C_{39}H_{80}N_2PO_6^-$	SM34:0(16:0) [M-H]$^-$	-121.2
71	704.41	$C_{38}H_{75}NPO_8^-$	PC30:0 [M-H]$^-$	-164.0
72	708.46	$C_{39}H_{67}NPO_8^-$	PE34:5 [M-H]$^-$	5.4
73	710.47	$C_{39}H_{69}NPO_8^-$	PE34:4 [M-H]$^-$	-9.3
74	712.51	$C_{39}H_{71}NPO_8^-$	PE34:3 [M-H]$^-$	21.0
75	713.46	$C_{40}H_{78}N_2PO_6^-$	SM36:2(18:2) [M-CH3]$^-$	-141.1
76	714.48	$C_{39}H_{73}NPO_8^-$	PE34:2 [M-H]$^-$	-41.1
77	715.51	$C_{40}H_{80}N_2PO_6^-$	SM36:1(18:1) [M-CH3]$^-$	-92.3
78	716.53	$C_{39}H_{75}NPO_8^-$	PE34:1 [M-H]$^-$	3.8
79	717.51	$C_{40}H_{82}N_2PO_6^-$	SM36:0(18:0) [M-CH3]$^-$	-117.5
80	718.35	$C_{41}H_{67}PO_8^-$	PC36:6 [M-TMA]$^-$	-155.0
81	718.68	$C_{39}H_{77}NPO_8^-$	PE34:0 [M-H]$^-$	201.0
82	720.47	$C_{41}H_{69}PO_8^-$	PC36:5 [M-TMA]$^-$	0.1
83	722.49	$C_{41}H_{71}PO_8^-$	PC36:4 [M-TMA]$^-$	-4.2

84	724.49	$C_{40}H_{71}NPO_8^-$	PC32:4 [M-H]$^-$	0.4
85	724.49	$C_{41}H_{73}PO_8^-$	PC36:3 [M-TMA]$^-$	-16.9
86	726.50	$C_{40}H_{73}NPO_8^-$	PC32:3 [M-H]$^-$	-11.4
87	726.50	$C_{41}H_{75}PO_8^-$	PC36:2 [M-TMA]$^-$	-28.7
88	727.50	$C_{41}H_{80}N_2PO_6^-$	SM36:2(18:2) [M-H]$^-$	-99.1
89	728.49	$C_{40}H_{75}NPO_8^-$	PC32:2 [M-H]$^-$	-43.9
90	728.49	$C_{41}H_{77}PO_8^-$	PC36:1 [M-TMA]$^-$	-61.1
91	729.50	$C_{41}H_{82}N_2PO_6^-$	SM36:1(18:1) [M-H]$^-$	-122.4
92	730.52	$C_{40}H_{77}NPO_8^-$	PC32:1 [M-H]$^-$	-19.5
93	730.52	$C_{41}H_{79}PO_8^-$	PC36:0 [M-TMA]$^-$	-36.7
94	731.44	$C_{41}H_{84}N_2PO_6^-$	SM36:0(18:0) [M-H]$^-$	-227.5
95	732.50	$C_{40}H_{79}NPO_8^-$	PC32:0 [M-H]$^-$	-79.3
96	734.47	$C_{41}H_{69}NPO_8^-$	PE36:6 [M-H]$^-$	-11.1
97	736.45	$C_{41}H_{71}NPO_8^-$	PE36:5 [M-H]$^-$	-55.1
98	738.45	$C_{41}H_{73}NPO_8^-$	PE36:4 [M-H]$^-$	-83.7
99	740.48	$C_{41}H_{75}NPO_8^-$	PE36:3 [M-H]$^-$	-53.5
100	742.46	$C_{41}H_{77}NPO_8^-$	PE36:2 [M-H]$^-$	-108.6
101	744.51	$C_{41}H_{79}NPO_8^-$	PE36:1 [M-H]$^-$	-64.5
102	746.58	$C_{41}H_{81}NPO_8^-$	PE36:0 [M-H]$^-$	7.8
103	750.46	$C_{42}H_{73}NPO_8^-$	PC34:5 [M-H]$^-$	-57.3
104	752.58	$C_{42}H_{75}NPO_8^-$	PC34:4 [M-H]$^-$	72.2
105	754.43	$C_{42}H_{77}NPO_8^-$	PC34:3 [M-H]$^-$	-141.3
106	756.49	$C_{42}H_{79}NPO_8^-$	PC34:2 [M-H]$^-$	-85.3
107	758.46	$C_{42}H_{81}NPO_8^-$	PC34:1 [M-H]$^-$	-143.8
108	760.51	$C_{42}H_{83}NPO_8^-$	PC34:0 [M-H]$^-$	-105.3
109	762.50	$C_{43}H_{73}NPO_8^-$	PE38:6 [M-H]$^-$	-4.7
110	764.50	$C_{43}H_{75}NPO_8^-$	PE38:5 [M-H]$^-$	-29.5
111	766.53	$C_{43}H_{77}NPO_8^-$	PE38:4 [M-H]$^-$	-7.5
112	768.59	$C_{43}H_{79}NPO_8^-$	PE38:3 [M-H]$^-$	39.5

113	770.43	$C_{43}H_{81}NPO_8^-$	PE38:2 [M-H]$^-$	-178.5
114	771.52	$C_{44}H_{88}N_2PO_6^-$	SM40:1(22:1) [M-CH3]$^-$	-160.0
115	772.41	$C_{43}H_{83}NPO_8^-$	PE38:1 [M-H]$^-$	-233.3
116	774.47	$C_{43}H_{85}NPO_8^-$	PE38:0 [M-H]$^-$	-169.5
117	776.60	$C_{44}H_{75}NPO_8^-$	PC36:6 [M-H]$^-$	97.8
118	778.47	$C_{44}H_{77}NPO_8^-$	PC36:5 [M-H]$^-$	-91.8
119	780.43	$C_{44}H_{79}NPO_8^-$	PC36:4 [M-H]$^-$	-165.2
120	782.56	$C_{44}H_{81}NPO_8^-$	PC36:3 [M-H]$^-$	-18.1
121	784.52	$C_{44}H_{83}NPO_8^-$	PC36:2 [M-H]$^-$	-83.7
122	786.47	$C_{44}H_{85}NPO_8^-$	PC36:1 [M-H]$^-$	-163.9
123	788.44	$C_{44}H_{87}NPO_8^-$	PC36:0 [M-H]$^-$	-221.6
124	790.39	$C_{45}H_{77}NPO_8^-$	PE40:6 [M-H]$^-$	-193.2
125	797.57	$C_{46}H_{90}N_2PO_6^-$	SM42:2(24:2) [M-CH3]$^-$	-106.1
126	799.73	$C_{46}H_{92}N_2PO_6^-$	SM42:1(24:1) [M-CH3]$^-$	73.2
127	803.31	$C_{41}H_{72}PO_{13}^-$	PI32:3 [M-H]$^-$	-199.7
128	805.32	$C_{41}H_{74}PO_{13}^-$	PI32:2 [M-H]$^-$	-208.9
129	807.51	$C_{41}H_{76}PO_{13}^-$	PI32:1 [M-H]$^-$	8.2
130	809.43	$C_{41}H_{78}PO_{13}^-$	PI32:0 [M-H]$^-$	-105.4
131	831.43	$C_{43}H_{76}PO_{13}^-$	PI34:3 [M-H]$^-$	-89.0
132	833.34	$C_{43}H_{78}PO_{13}^-$	PI34:2 [M-H]$^-$	-211.8
133	835.58	$C_{43}H_{80}PO_{13}^-$	PI34:1 [M-H]$^-$	53.5
134	837.52	$C_{43}H_{82}PO_{13}^-$	PI34:0 [M-H]$^-$	-34.4
135	855.63	$C_{45}H_{76}PO_{13}^-$	PI36:5 [M-H]$^-$	144.4
136	857.59	$C_{45}H_{78}PO_{13}^-$	PI36:4 [M-H]$^-$	85.4
137	859.52	$C_{45}H_{80}PO_{13}^-$	PI36:3 [M-H]$^-$	-19.8
138	861.55	$C_{45}H_{82}PO_{13}^-$	PI36:2 [M-H]$^-$	5.4
139	863.57	$C_{45}H_{84}PO_{13}^-$	PI36:1 [M-H]$^-$	4.8
140	865.59	$C_{45}H_{86}PO_{13}^-$	PI36:0 [M-H]$^-$	13.0
141	885.48	$C_{47}H_{82}PO_{13}^-$	PI38:4 [M-H]$^-$	-73.6

142	887.55	$C_{47}H_{84}PO_{13}^-$	PI38:3 [M-H]⁻	-17.6
143	909.56	$C_{49}H_{82}PO_{13}^-$	PI40:6 [M-H]⁻	9.6
144	911.56	$C_{49}H_{84}PO_{13}^-$	PI40:5 [M-H]⁻	-7.5
145	913.58	$C_{49}H_{86}PO_{13}^-$	PI40:4 [M-H]⁻	-3.4

Table S2. Peak assignment of ToF-SIMS spectra of cardiomyocytes to corresponding positive ions.

No.	Center Mass (u)	Assignment	Description	Mass Deviation (ppm)
1	237.24	$C_{16}H_{29}O^+$	FA16:1 $[M-H2O]^+$	59.5
2	239.23	$C_{16}H_{31}O^+$	FA16:0 $[M-H2O]^+$	-21.5
3	255.26	$C_{16}H_{31}O_2^+$	FA16:1 $[M+H]^+$	92.8
4	257.23	$C_{16}H_{33}O_2^+$	FA16:0 $[M+H]^+$	-54.0
5	263.24	$C_{18}H_{31}O^+$	FA18:2 $[M-H2O]^+$	8.6
6	265.27	$C_{18}H_{33}O^+$	FA18:1 $[M-H2O]^+$	74.8
7	267.29	$C_{18}H_{35}O^+$	FA18:0 $[M-H2O]^+$	77.2
8	279.27	$C_{18}H_{31}O_2^+$	FA18:3 $[M+H]^+$	143.1
9	281.27	$C_{18}H_{33}O_2^+$	FA18:2 $[M+H]^+$	80.6
10	283.24	$C_{18}H_{35}O_2^+$	FA18:1 $[M+H]^+$	-70.4
11	285.29	$C_{18}H_{37}O_2^+$	FA18:0 $[M+H]^+$	23.5
12	291.30	$C_{20}H_{35}O^+$	FA20:2 [M-H2O]+	116.0
13	295.33	$C_{20}H_{39}O^+$	FA20:0 $[M-H2O]^+$	98.8
14	307.27	$C_{20}H_{35}O_2^+$	FA20:3 $[M+H]^+$	27.9
15	309.26	$C_{20}H_{37}O_2^+$	FA20:2 $[M+H]^+$	-73.2
16	309.26	$C_{19}H_{33}O_3^+$	MAG16:2 $[M-OH]^+$	44.5
17	311.27	$C_{20}H_{39}O_2^+$	FA20:1 $[M+H]^+$	-83.7
18	311.27	$C_{19}H_{35}O_3^+$	MAG16:1 $[M-OH]^+$	33.1
19	313.29	$C_{20}H_{41}O_2^+$	FA20:0 $[M+H]^+$	-78.4
20	313.29	$C_{19}H_{37}O_3^+$	MAG16:0 $[M-OH]^+$	37.7

21	337.27	$C_{21}H_{37}O_3^+$	MAG18:2 [M-OH]$^+$	2.4
22	339.27	$C_{21}H_{39}O_3^+$	MAG18:1 [M-OH]$^+$	-42.6
23	341.31	$C_{21}H_{41}O_3^+$	MAG18:0 [M-OH]$^+$	6.4
24	367.31	$C_{23}H_{43}O_3^+$	MAG20:2 [M-OH]$^+$	-38.9
25	369.33	$C_{27}H_{45}^+$	Cholesterol [M-OH]$^+$	-69.3
26	371.36	$C_{23}H_{47}O_3^+$	MAG20:0 [M-OH]$^+$	10.1
27	385.35	$C_{27}H_{45}O^+$	Cholesterol	19.1
28	493.47	$C_{31}H_{57}O_4^+$	DAG28:1 [M-OH]$^+$	97.7
29	495.55	$C_{31}H_{59}O_4^+$	DAG28:0 [M-OH]$^+$	211.1
30	517.50	$C_{33}H_{57}O_4^+$	DAG30:3 [M-OH]$^+$	140.7
31	519.49	$C_{33}H_{59}O_4^+$	DAG28:2 [M-OH]$^+$	91.3
32	521.45	$C_{33}H_{61}O_4^+$	DAG30:1 [M-OH]$^+$	-4.1
33	523.53	$C_{33}H_{63}O_4^+$	DAG30:0 [M-OH]$^+$	108.3
34	533.49	$C_{31}H_{58}O_5Na^+$	DAG28:1 [M+Na]$^+$	144.2
35	535.53	$C_{31}H_{60}O_5Na^+$	DAG28:0 [M+Na]$^+$	171.3
36	543.56	$C_{35}H_{59}O_4^+$	DAG32:4 [M-OH]$^+$	216.6
37	545.44	$C_{35}H_{61}O_4^+$	DAG32:3 [M-OH]$^+$	-28.3
38	547.32	$C_{35}H_{63}O_4^+$	DAG32:2 [M-OH]$^+$	-280.7
39	549.53	$C_{35}H_{65}O_4^+$	DAG32:1 [M-OH]$^+$	69.1
40	551.48	$C_{35}H_{67}O_4^+$	DAG32:0 [M-OH]$^+$	-37.9
41	557.53	$C_{33}H_{58}O_5Na^+$	DAG30:3 [M+Na]$^+$	208.5
42	559.53	$C_{33}H_{60}O_5Na^+$	DAG30:2 [M+Na]$^+$	170.8
43	561.52	$C_{33}H_{62}O_5Na^+$	DAG30:1 [M+Na]$^+$	120.8
44	563.65	$C_{33}H_{64}O_5Na^+$	DAG30:0 [M+Na]$^+$	335.8
45	571.44	$C_{37}H_{63}O_4^+$	DAG34:4 [M-OH]$^+$	-63.8
46	573.60	$C_{37}H_{65}O_4^+$	DAG34:3 [M-OH]$^+$	190.8
47	575.44	$C_{37}H_{67}O_4^+$	DAG34:2 [M-OH]$^+$	-104.2
48	577.45	$C_{37}H_{69}O_4^+$	DAG34:1 [M-OH]$^+$	-127.0
49	579.58	$C_{37}H_{71}O_4^+$	DAG34:0 [M-OH]$^+$	72.7

50	585.55	$C_{35}H_{62}O_5Na^+$	DAG32:3 $[M+Na]^+$	174.8
51	587.53	$C_{35}H_{64}O_5Na^+$	DAG32:2 $[M+Na]^+$	115.8
52	589.53	$C_{35}H_{66}O_5Na^+$	DAG32:1 $[M+Na]^+$	90.1
53	591.50	$C_{35}H_{68}O_5Na^+$	DAG32:0 $[M+Na]^+$	6.3
54	597.60	$C_{39}H_{65}O_4^+$	DAG36:5 $[M-OH]^+$	188.1
55	599.42	$C_{39}H_{67}O_4^+$	DAG36:4 $[M-OH]^+$	-138.9
56	601.44	$C_{39}H_{69}O_4^+$	DAG36:3 $[M-OH]^+$	-133.4
57	603.47	$C_{39}H_{71}O_4^+$	DAG36:2 $[M-OH]^+$	-105.4
58	605.45	$C_{39}H_{73}O_4^+$	DAG36:1 $[M-OH]^+$	-163.1
59	607.72	$C_{39}H_{75}O_4^+$	DAG36:0 $[M-OH]^+$	248.6
60	615.48	$C_{37}H_{68}O_5Na^+$	DAG34:2 $[M+Na]^+$	-20.9
61	617.47	$C_{37}H_{70}O_5Na^+$	DAG34:1 $[M+Na]^+$	-60.4
62	619.49	$C_{37}H_{72}O_5Na^+$	DAG34:0 $[M+Na]^+$	-68.0
63	625.51	$C_{41}H_{69}O_4^+$	DAG38:5 $[M-OH]^+$	-11.8
64	627.71	$C_{41}H_{71}O_4^+$	DAG38:4 $[M-OH]^+$	284.3
65	629.62	$C_{41}H_{73}O_4^+$	DAG38:3 $[M-OH]^+$	117.2
66	631.52	$C_{41}H_{75}O_4^+$	DAG38:2 $[M-OH]^+$	-78.7
67	632.50	$C_{33}H_{63}NPO_8^+$	PE28:2 $[M+H]^+$	115.1
68	633.58	$C_{41}H_{77}O_4^+$	DAG38:1 $[M-OH]^+$	-3.9
69	635.68	$C_{41}H_{79}O_4^+$	DAG38:0 $[M-OH]^+$	129.1
70	656.25	$C_{33}H_{64}NPO_8Na^+$	PE28:1 $[M+Na]^+$	-260.9
71	658.38	$C_{33}H_{66}NPO_8Na^+$	PE28:0 $[M+Na]^+$	-97.9
72	658.38	$C_{35}H_{65}NPO_8^+$	PE30:3 $[M+H]^+$	-101.6
73	660.48	$C_{35}H_{67}NPO_8^+$	PE30:2 $[M+H]^+$	23.1
74	662.45	$C_{35}H_{69}NPO_8^+$	PE30:1 $[M+H]^+$	-44.3
75	664.31	$C_{35}H_{71}NPO_8^+$	PE30:0 $[M+H]^+$	-279.8
76	676.46	$C_{36}H_{71}NPO_8^+$	PC28:1 $[M+H]^+$	-47.0
77	680.32	$C_{35}H_{64}NPO_8Na^+$	PE30:3 $[M+Na]^+$	-153.9
78	682.36	$C_{35}H_{66}NPO_8Na^+$	PE30:2 $[M+Na]^+$	-114.0

79	684.37	$C_{35}H_{68}NPO_8Na^+$	PE30:1 $[M+Na]^+$	-127.0
80	684.37	$C_{37}H_{67}NPO_8^+$	PE32:4 $[M+H]^+$	-130.5
81	686.35	$C_{35}H_{70}NPO_8Na^+$	PE30:0 $[M+Na]^+$	-177.9
82	686.35	$C_{37}H_{69}NPO_8^+$	PE32:3 $[M+H]^+$	-181.4
83	688.54	$C_{37}H_{71}NPO_8^+$	PE32:2 $[M+H]^+$	67.5
84	698.49	$C_{36}H_{70}NPO_8Na^+$	PC28:1 $[M+Na]^+$	27.5
85	698.49	$C_{38}H_{69}NPO_8^+$	PC30:4 $[M+H]^+$	24.1
86	700.38	$C_{36}H_{72}NPO_8Na^+$	PC28:0 $[M+Na]^+$	-162.2
87	700.38	$C_{38}H_{71}NPO_8^+$	PC30:3 $[M+H]^+$	-165.6
88	702.47	$C_{38}H_{73}NPO_8^+$	PC30:2 $[M+H]^+$	-56.1
89	704.48	$C_{38}H_{75}NPO_8^+$	PC30:1 $[M+H]^+$	-64.4
90	710.38	$C_{37}H_{70}NPO_8Na^+$	PE32:2 $[M+Na]^+$	-124.7
91	710.38	$C_{39}H_{69}NPO_8^+$	PE34:5 $[M+H]^+$	-128.1
92	712.31	$C_{37}H_{72}NPO_8Na^+$	PE32:1 $[M+Na]^+$	-248.1
93	712.31	$C_{39}H_{71}NPO_8^+$	PE34:4 $[M+H]^+$	-251.4
94	714.51	$C_{39}H_{73}NPO_8^+$	PE34:3 $[M+H]^+$	6.7
95	716.46	$C_{39}H_{75}NPO_8$+	PE34:2 $[M+H]^+$	-82.8
96	718.45	$C_{39}H_{77}NPO_8^+$	PE34:1 $[M+H]^+$	-122.4
97	724.40	$C_{38}H_{72}NPO_8Na^+$	PC30:2 $[M+Na]^+$	-116.3
98	726.50	$C_{38}H_{74}NPO_8Na^+$	PC30:1 $[M+Na]^+$	-1.5
99	726.50	$C_{40}H_{73}NPO_8^+$	PC32:4 $[M+H]^+$	-4.8
100	728.38	$C_{38}H_{76}NPO_8Na^+$	PC30:0 $[M+Na]^+$	-197.9
101	728.38	$C_{40}H_{75}NPO_8^+$	PC32:3 $[M+H]^+$	-201.3
102	730.29	$C_{40}H_{77}NPO_8^+$	PC32:2 $[M+H]^+$	-340.2
103	732.67	$C_{40}H_{79}NPO_8^+$	PC32:1 $[M+H]^+$	153.9
104	732.67	$C_{39}H_{68}NPO_8Na^+$	PE34:5 $[M+Na]^+$	285.4
105	734.45	$C_{40}H_{81}NPO_8^+$	PC32:0 $[M+H]^+$	-156.3
106	734.45	$C_{39}H_{70}NPO_8Na^+$	PE34:4 $[M+Na]^+$	-25.2
107	740.41	$C_{39}H_{76}NPO_8Na^+$	PE34:1 $[M+Na]^+$	-151.4

108	740.41	$C_{41}H_{75}NPO_8^+$	PE36:4 $[M+H]^+$	-154.6
109	742.54	$C_{39}H_{78}NPO_8Na^+$	PE34:0 $[M+H]^+$	4.2
110	742.54	$C_{41}H_{77}NPO_8^+$	PE36:3 $[M+H]^+$	0.9
111	744.49	$C_{41}H_{79}NPO_8^+$	PE36:2 $[M+H]^+$	-83.7
112	746.40	$C_{41}H_{81}NPO_8^+$	PE35:1 $[M+H]^+$	-223.5
113	754.53	$C_{42}H_{77}NPO_8^+$	PC34:4 $[M+H]^+$	-6.5
114	756.52	$C_{40}H_{80}NPO_8Na^+$	PC32:0 $[M+Na]^+$	-47.3
115	756.52	$C_{42}H_{79}NPO_8^+$	PC34:3 $[M+H]^+$	-50.5
116	758.60	$C_{42}H_{81}NPO_8^+$	PC34:2 $[M+H]^+$	41.7
117	760.43	$C_{42}H_{83}NPO_8^+$	PC34:1 $[M+H]^+$	-202.4
118	762.60	$C_{42}H_{85}NPO_8^+$	PC34:0 $[M+H]^+$	-0.3
119	762.60	$C_{41}H_{74}NPO_8Na^+$	PE36:4 $[M+Na]^+$	126.0
120	764.42	$C_{41}H_{76}NPO_8Na^+$	PE36:3 $[M+Na]^+$	-136.7
121	766.60	$C_{41}H_{78}NPO_8Na^+$	PE36:2 $[M+Na]^+$	80.2
122	768.34	$C_{41}H_{80}NPO_8Na^+$	PE36:1 $[M+Na]^+$	-278.3
123	770.37	$C_{41}H_{82}NPO_8Na^+$	PE36:0 $[M+Na]^+$	-252.7
124	780.44	$C_{42}H_{80}NPO_8Na^+$	PC34:2 $[M+Na]^+$	-141.8
125	780.44	$C_{44}H_{79}NPO_8^+$	PC36:5 $[M+H]^+$	-144.9
126	782.49	$C_{42}H_{82}NPO_8Na^+$	PC34:1 $[M+Na]^+$	-96.0
127	782.49	$C_{44}H_{81}NPO_8^+$	PC36:4 $[M+H]^+$	-99.1
128	784.47	$C_{42}H_{84}NPO_8Na^+$	PC34:0 $[M+Na]^+$	-138.8
129	784.47	$C_{44}H_{83}NPO_8^+$	PC36:3 $[M+H]^+$	-141.9
130	786.58	$C_{44}H_{85}NPO_8^+$	PC36:2 $[M+H]^+$	-27.7
131	788.46	$C_{44}H_{87}NPO_8^+$	PC36:1 $[M+H]^+$	-193.9
132	796.57	$C_{43}H_{84}NPO_8Na^+$	PE38:1 $[M+Na]^+$	-11.2
133	798.58	$C_{43}H_{86}NPO_8Na^+$	PE38:0 $[M+Na]^+$	-22.8
134	804.58	$C_{44}H_{80}NPO_8Na^+$	PC36:4 $[M+Na]^+$	31.9
135	804.58	$C_{46}H_{79}NPO_8^+$	PC38:7 $[M+H]^+$	28.9
136	806.57	$C_{44}H_{82}NPO_8Na^+$	PC36:3 $[M+Na]^+$	9.6

137	806.57	$C_{46}H_{81}NPO_8^+$	PC38:6 $[M+H]^+$	6.6
138	808.51	$C_{44}H_{84}NPO_8Na^+$	PC36:2 $[M+Na]^+$	-88.4
139	808.51	$C_{46}H_{83}NPO_8^+$	PC38:5 $[M+H]^+$	-91.4
140	810.47	$C_{44}H_{86}NPO_8Na^+$	PC36:1 $[M+Na]^+$	-155.8
141	810.47	$C_{46}H_{85}NPO_8^+$	PC38:4 $[M+H]^+$	-158.8
142	812.43	$C_{44}H_{88}NPO_8Na^+$	PC36:0 $[M+Na]^+$	-228.3
143	819.56	$C_{51}H_{88}O_6Na^+$	TAG48:5 $[M+Na]^+$	-110.7
144	821.51	$C_{51}H_{90}O_6Na^+$	TAG48:4 $[M+Na]^+$	-180.7
145	823.88	$C_{51}H_{92}O_6Na^+$	TAG48:3 $[M+Na]^+$	249.6
146	825.82	$C_{51}H_{94}O_6Na^+$	TAG48:2 $[M+Na]^+$	157.3
147	826.58	$C_{46}H_{78}NPO_8Na^+$	PC38:7 $[M+Na]^+$	58.6
148	827.76	$C_{51}H_{96}O_6Na^+$	TAG48:1 $[M+Na]^+$	58.7
149	828.53	$C_{46}H_{80}NPO_8Na^+$	PC38:6 $[M+Na]^+$	-24.0
150	829.82	$C_{51}H_{98}O_6Na^+$	TAG48:0 $[M+Na]^+$	112.9
151	830.68	$C_{46}H_{82}NPO_8Na^+$	PC38:5 $[M+Na]^+$	140.6
152	832.47	$C_{46}H_{84}NPO_8Na^+$	PC38:4 $[M+Na]^+$	-129.9
153	834.55	$C_{46}H_{86}NPO_8Na^+$	PC38:3 $[M+Na]^+$	-62.3
154	836.59	$C_{46}H_{88}NPO_8Na^+$	PC38:2 $[M+Na]^+$	-32.3
155	849.34	$C_{53}H_{94}O_6Na^+$	TAG50:4 $[M+Na]^+$	-422.3
156	851.49	$C_{53}H_{96}O_6Na^+$	TAG50:3 $[M+Na]^+$	-263.3
157	853.50	$C_{53}H_{98}O_6Na^+$	TAG50:2 $[M+Na]^+$	-265.6
158	855.56	$C_{53}H_{100}O_6Na^+$	TAG50:1 $[M+Na]^+$	-211.9
159	857.85	$C_{53}H_{102}O_6Na^+$	TAG50:0 $[M+Na]^+$	114.0
160	880.04	$C_{55}H_{100}O_6Na^+$	TAG52:3 $[M+Na]^+$	344.2
161	881.66	$C_{55}H_{102}O_6Na^+$	TAG52:2 $[M+Na]^+$	-108.0
162	883.86	$C_{55}H_{104}O_6Na^+$	TAG52:1 $[M+Na]^+$	95.4
163	885.81	$C_{55}H_{106}O_6Na^+$	TAG52:0 $[M+Na]^+$	22.6
164	907.85	$C_{57}H_{104}O_6Na^+$	TAG54:3 $[M+Na]^+$	87.4
165	909.98	$C_{57}H_{106}O_6Na^+$	TAG54:2 $[M+Na]^+$	206.0

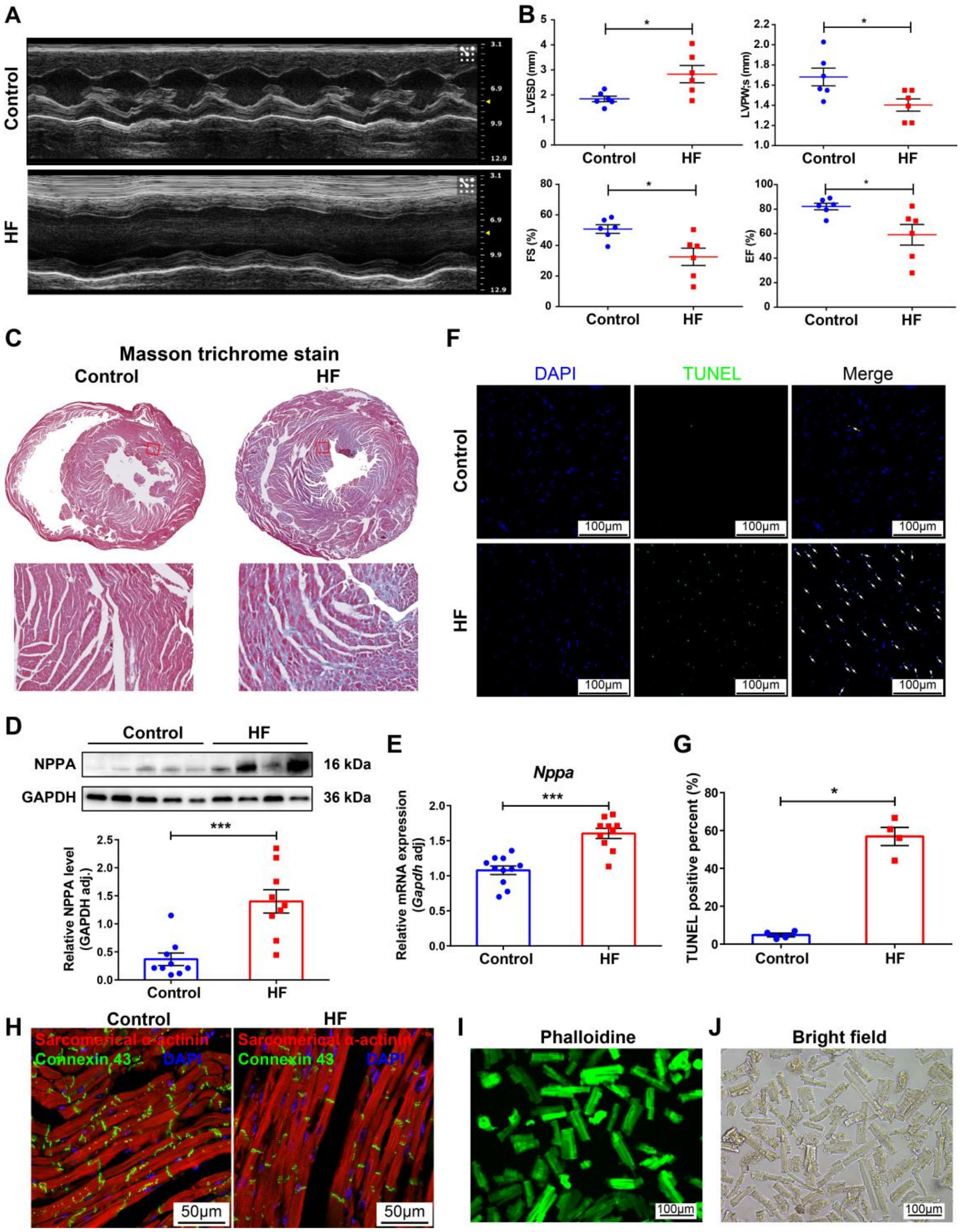

Figure S1. Characterization of the heart failure mouse model

(A) Representative echocardiographic images of healthy control(upper) and HF hearts (lower). (B) M-mode echocardiography of cardiac morphology and function in control and HF mice: No statistically significant difference was observed between mice about the heart weight; while HF mice demonstrated typical dilation of the heart

(LVESD), thinning ventricular walls (LVPW), and impaired ventricular systolic function (FS, EF). (C) Masson's-Trichrome staining of HF mice showed significantly increase in cardiac fibrosis. (D) Representative western blot analysis and quantification of NPPA in the myocardium of control and HF mice. (E) of the difference in the transcription of *Nppa* mRNA in cardiac tissue of control and HF mice; *Gapdh* was used as the reference gene). (F) Representative immunofluorescence images of TUNEL staining (green). TUNEL positive cells are indicated by the white arrows. Scale bar = 100 μm. (G) Percentage of TUNEL positive cell in myocardium of control and HF mice. (H) Inmmunofluorescence staining with connexin 43 (green) and sarcomerical α-actinin (red). The morphology and cell junctions of cardiomyocyte were impaired, which could be evidenced by the abnormal Cx43 staining (reduced and ectopic expression) in HF myocardium. (I) Immunofluorescence staining with phalloidine (green, visualizing the myofibers/ actin cytoskeleton) of freshly isolated mouse cardiomyocytes. (J) Bright-field microscopy images on the freshly isolated mouse cardiomyocytes.

HF, heart failure; LVESD, left ventricular end systolic diameter; LVPW, left ventricular posterior wall thickness; FS, fractional shortening; EF, left ventricular ejection fraction; TUNEL, terminal-deoxynucleoitidyl transferase mediated nick end labeling. All of the data represented as mean ± SEM; $^{*}P < 0.05$, $^{**}P < 0.01$, $^{***}P < 0.001$.

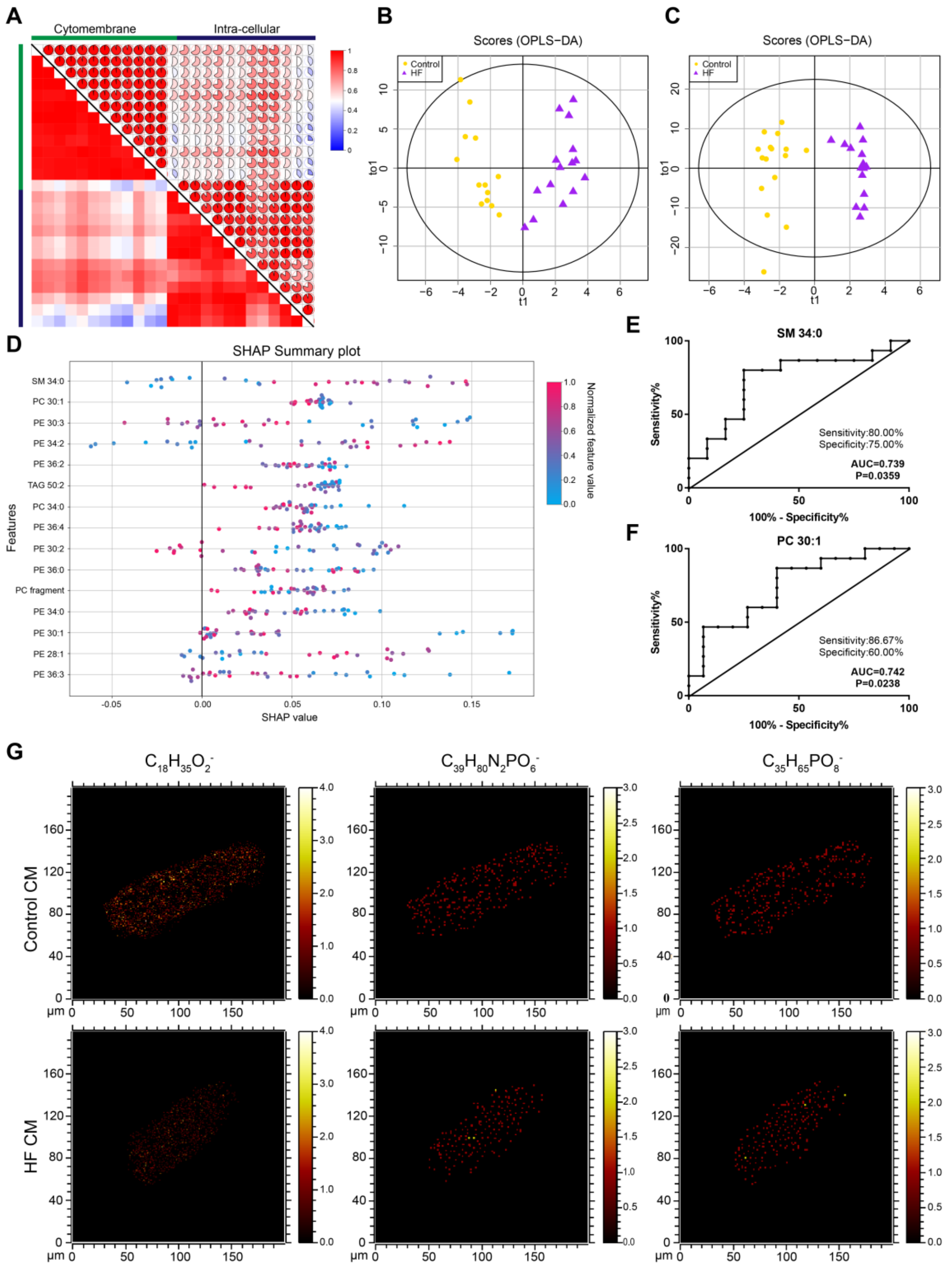

Figure S2. **Differential metabolomic analysis of the cardiomyocytes.** (A) The expressions of detected metabolites were significantly different between the cytomembrane and the intracellular in the positive ion mass spectra. (B-C) OPLS-DA presented stark differences between the metabolites of the intracellular of two groups (HF VS. C): (B) for the negative ion mass spectra, and (C) for the positive ion mass spectra. (D) SHAP plot displays variables in a top-to-bottom format to demonstrate

the feature importance for prediction of heart failure in intracellular. (E-F) Receiver operating characteristic curve to quantify the discriminability of metabolites to distinguish between the two groups (HF VS. C). (G) ToF-SIMS imaging of cardiomocytes intracellular of control and HF. $C_{18}H_{35}O_2^-$ is a characteristic fragment of FA 18:0, $C_{39}H_{80}N_2PO_6^-$ is a characteristic fragment of SM 34:0 and $C_{35}H_{65}PO_8^-$ is a characteristic fragment of PC 30:1. OPLS-DA, orthogonal partial least squares-discriminant analysis; HF, heart failure; SHAP, SHapley Additive exPlanation.

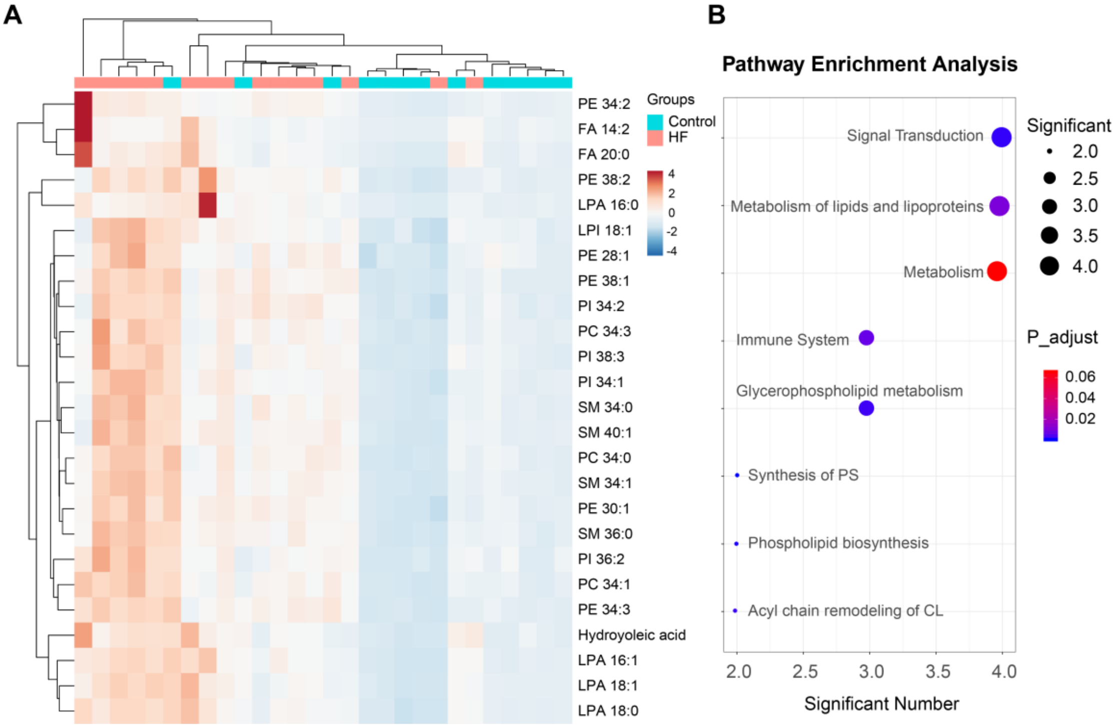

Figure S3. Functional implications of intracellular differential metabolites of cardiomyocytes. (A) Hierarchical cluster analysis was used to visualize the clustering and relevance of the differential metabolites. (B) Pathway enrichment analysis of these differential metabolites. HF, heart failure.
